# Effects of dietary postbiotic and inulin on growth performance, IGF1 and GHR mRNA expression, faecal microbiota and volatile fatty acids in broilers

**DOI:** 10.1186/s12917-016-0790-9

**Published:** 2016-08-05

**Authors:** Karwan Yaseen Kareem, Teck Chwen Loh, Hooi Ling Foo, Henny Akit, Anjas Asmara Samsudin

**Affiliations:** 1 Department of Animal Science, Faculty of Agriculture, Universiti Putra Malaysia, 43400 UPM, Serdang, Selangor Malaysia; 2 Institute of Tropical Agriculture, Universiti Putra Malaysia, 43400 UPM, Serdang, Selangor Malaysia; 3 Department of Bioprocess Technology, Faculty of Biotechnology and Biomolecular Science, Universiti Putra Malaysia, 43400 UPM, Serdang, Selangor Malaysia; 4 Institute of Bioscience, Universiti Putra Malaysia, 43400 UPM, Serdang, Selangor Malaysia; 5 Department of Animal Resource, University of salah al-Din, Erbil, Iraq

**Keywords:** Broilers, Inulin, Prebiotic, Postbiotic, Intestinal microbiota, IGF1, GHR, Volatile fatty acid

## Abstract

**Background:**

Postbiotics (metabolic products by lactic acid bacteria) and prebiotics have been established as substitute to antibiotics in order to enhance immunity and growth performance in broiler chickens. Nonetheless, insufficient information is available on the effects of postbiotics and prebiotics combination on growth performance, faecal microbiota, pH and volatile fatty acids (VFA), as well as liver insulin like growth factor 1 (IGF1) and growth hormone receptor (GHR) mRNA expressions in broiler chickens. The aim of this experiment was to evaluate the effects of different types of postbiotics with different levels of prebiotic (inulin) on broiler for those parameters.

**Results:**

The results showed that birds fed T3: (0.3 % RI11 + 0.8 % Inulin), T4: (0.3 % RI11 + 1.0 % Inulin), and T6: (0.3 % RG14+ 1.0 % Inulin) had higher (*p* < 0.05) final body weight (BW) and total weight gain (WG) than other treatments. Birds fed T3 had lower feed conversion ratio (FCR) which was significantly different from those fed with negative control diet but was similar to other treatments. Postbiotic and inulin increased (*p* < 0.05) faecal lactic acid bacteria (LAB) and reduced (*p* < 0.05) *Enterobacteriaceae* count. Birds fed T4 and T6 had higher faecal acetic acid and propionic acid respectively, and both had higher total VFA and lactic acid bacteria but lower pH and *Enterobacteriaceae* (ENT) counts compared to other treatments. The liver of birds fed T4 and T6 had higher IGF1 expression compared to other treatments while T6 had higher GHR mRNA expression compared to other treatments.

**Conclusions:**

Results indicate that the addition of postbiotics and inulin combinations had beneficial effects on total BW, feed efficiency, mucosa architecture and IGF1 and GHR mRNA expression in broiler chickens.

## Background

Intestinal microbiota play a vital role in the nutritional, physiological, immunological, and protective functions of the host [1] and their composition and activities can be influenced by diet [2]. The efficacy of feeding sub-therapeutic levels of antibiotics to modulate gut microbiota to enhance production performance of livestock has been espoused [3]. Unfortunately, the usage of antibiotics as feed additives for long periods in poultry diets can lead to antibiotic resistance [4] and high residue levels in poultry products such as meat and egg [5, 6]. Antimicrobial resistance encoding genes may represent risk to both human and animal health if it is transferred to other formerly susceptible bacteria [7]. Since the quest for safer and healthier chicken meat has remarkably increased in recent time, the use of natural feed additives can produce antibiotic-free chicken and can also prevent foodborne diseases [8].

In recent years, several feed additives such as prebiotics, probiotics, symbiotics, postbiotics and the combination of postbiotics and prebiotics have been used as growth promoters to replace antibiotics [9–12]. The mode of action of these additives differs. Probiotics colonize the host digestive system, increase the natural microbiota and prevent the colonization of pathogenic organisms [10]. Despite their beneficial effects, most probiotics especially the plasmids probiotics have antibiotic resistance genes which can be transferred between organisms [13]. As a consequence, probiotic as a live bacteria might not be used anymore in the near future. As a substitute to probiotics, metabolite products synthesized from probiotic known as postbiotics could be used. It is believed that postbiotics have the probiotic effects without living cells [14–16]. Prebiotics are non-living fibrous feed additives which when added to feed are preferred by harmful microbes. Prebiotics control the growth of pathogens (i.e. *Escherichia coli* and *Salmonella*) and stimulate the growth of *Bifidobacteria* and *Lactobacilli* and consequently promoting the health and performance of animals [17, 18]. A typical example of prebiotics is inulin. Postbiotics and inulin combination inhibited reproduction of pathogenic bacteria such as *Listeria monocytogenes, Salmonella enterica, Escherichia coli* and Vancomysin Resistant *Enterococci* [19]. Furthermore, addition of metabolite combinations to the feed of broilers [14, 15], laying hens [20] and pigs [16] improved the growth performance, faecal lactic acid bacteria and villus height. Various studies have examined the effects of postbiotic and prebiotics on growth performance, intestinal microbial ecology and histomorphology of broilers. However, there is dearth of information on the use of postbiotics and prebiotics combination and their synergistic effects on growth performance, intestinal microbial ecology, faecal VFA and histomorphology.

Chicken IGF1 has been identified as a biological candidate gene responsible for body composition, growth, fat deposition and metabolic activities in chickens [21]. It has been reported that the IGF-I level, feeding level, and growth rate are concurrent [22]. The dependence of nutritional and growth hormones on hepatic IGF1 production has been demonstrated [23, 24]. The pituitary releases the growth hormone which stimulates the hepatic production of IGF1 through the actions of GH activated GH receptors. However, the overall nutritional status of the animal modulates the ability of hepatic tissue to respond to GH. The IGF1 level can be affected by factors and situations that affect primary processes and controll the IGF1 production [25]. The GHR gene play vital role as a mediator of body size in bird [26, 27]. Since probiotics, prebiotics, antiobiotics and postbiotics influenced growth peroformance in poultry, a relationship between the feed additives and genes related to growth is anticipated. Thus, the aim of this work was to examine the effect of postbiotics and prebiotics on growth performance, IGF1 and GHR expression, intestinal microbial ecology, histomorphology and faecal VFA in broilers.

## Methods

### Postbiotics and inulin

The stock culture of *Lactobacillus plantarum* (*L. plantarum*) RG14 and *L. plantarum* RI11 were prepared at the Laboratory of Prebiotic and Probiotic Technology II at Institute of Bioscience, Universiti Putra Malaysia. The stock cultures were revived two times using de-Mann Rogosa Sharpe (MRS) broth and incubated at 30 °C for 48 and 24 h subsequently at static condition, followed by spread plate and incubation was performed in 48 h at 30 °C. A single colony was then picked and inoculated into 10 mL MRS broth and incubated for 24 h. It was followed by subculturing it into 10 mL MRS broth and incubated for 24 h at 30 °C. The culture was then ready to be used as an inoculum. An inoculum size of 1 % (v/v) was inoculated into the respective reconstituted media and incubated for 24 h at 30 °C at static condition. Centrifugation at 10,000 × g for 15 min was performed to separate the bacterial cell. The postbiotics were collected and kept at 4 °C [28] prior to feeding trials. The inulin (Frutafit IQ) was provided by Connell Bros. Company (Malaysia) Sdn. Bhd.

### Animals and experimental design

Two hundred and eighty-eight day old chicks were purchased from a commercial hatchery. The broiler chickens were allocated into eight treatment groups. Each group had six replicates while each replicate had six birds. The treatment groups included basal diet (negative control), basal diet + neomycin and oxytetracycline (positive control), T1 = Basal diet + 0.3 % postbiotic RI11, T2 = Basal diet + 0.3 % postbiotic RG14, T3 = Basal diet + 0.3 % postbiotic RI11 + 0.8 % inulin, T4 = Basal diet + 0.3 % postbiotic RI11 + 1.0 % inulin, T5 = Basal diet + 0.3 % postbiotic RG14 + 0.8 % inulin, T6 = Basal diet + 0.3 %, postbiotic RG14 + 1.0 % inulin. Water and feed were offered ad libitum to the birds until 42 days of age. Starter and finisher diets (Tables 1 and 2) were offered from days 0 to 21 and days 22 to 42, respectively. The experimental animals received humane care as outlined and approved by Institutional Animal Care and Use Committee for the Care and Use of Animals for Scientific Purposes (Research Policy, Universiti Putra Malaysia).

**Table 1:** Composition and nutrient content of starter diets

Ingredients	Dietary treatment^a^							
	Negative control	Positive control	T1	T2	T3	T4	T5	T6
Corn	50.00	50.00	50.18	50.18	50.20	50.20	50.20	50.20
Soybean	29.380	29.375	30.94	30.94	29.995	30.00	29.995	30.00
Wheat pollard	6.895	6.895	4.645	4.645	4.820	4.490	4.820	4.490
CPO	3.400	3.400	3.680	3.680	3.590	3.665	3.590	3.665
Fish meal (55 %)	7.580	7.575	6.825	6.825	7.550	7.600	7.550	7.600
L-Lysine	0.25	0.25	0.25	0.25	0.25	0.25	0.25	0.25
DL-Methionine	0.20	0.20	0.15	0.15	0.20	0.20	0.20	0.20
Monodicalcium phosphate21	1.00	1.00	1.10	1.10	1.00	1.00	1.00	1.00
Calcium carbonate	0.68	0.68	1.0	1.0	0.68	0.68	0.68	0.68
Choline chloride	0.06	0.06	0.06	0.06	0.06	0.06	0.06	0.06
Salt	0.25	0.25	0.30	0.30	0.25	0.25	0.25	0.25
Mineral premix^b^	0.10	0.10	0.10	0.10	0.10	0.10	0.10	0.10
Vitamin premix^c^	0.06	0.06	0.06	0.06	0.06	0.06	0.06	0.06
Antioxidant^d^	0.01	0.01	0.01	0.01	0.01	0.01	0.01	0.01
Toxin binder^e^	0.135	0.135	0.400	0.400	0.135	0.135	0.135	0.135
Antibiotic^f^		0.01						
postbiotic RI11			0.30		0.30	0.30		
postbiotic RG14				0.30			0.30	0.30
Inulin					0.80	1.00	0.80	1.00
Calculated nutrient content (g/kg)^g^								
Crude protein	220.5	220.4	220.0	220.0	220.4	220.3	220.4	220.3
Metabolizable energy (MJ/Kg)	12.96	12.96	12.96	12.96	12.95	12.95	12.95	12.95
Calcium	9.9	9.9	10.01	10.01	9.9	9.9	9.9	9.9
Total phosphorus	8.5	8.5	8.3	8.3	8.5	8.3	8.3	8.3
Avail. P for poultry	5.1	5.1	5.1	5.1	5.1	5.1	5.1	5.1

^a^Negative control: (basal diet), Positive control: (basal diet + neomycin and oxytetracycline), T1: (0.3 % RI11), T2: (0.3 % RG14), T3: (0.3 % RI11 + 0.8 % Inulin), T4: (0.3 % RI11 + 1.0 % Inulin), T5: (0.3 % RG14 + 0.8 % Inulin), T6: (0.3 % RG14+ 1.0 % Inulin)

^b^Mineral mix contains Fe 100 mg, Mn 110 mg, Cu 20 mg, Zn 100 mg, I 2 mg, Se 0.2 mg, Co 0.6 mg

^c^Vitamin premix contains retinol 2 mg, cholicalciferol 0.03 mg, α-tocopherol 0.02 mg, menadione 1.33 mg, cobalamin 0.03 mg, thiamine 0.83 mg, riboflavin 2 mg, folic acid 0.33 mg, biotin 0.03 mg, panthothenic acid 3.75 mg, niacin 23.3 mg, pyridoxine 1.33 mg

^d^Antioxidant contains butylated hydroxyanisole (BHA)

^e^Toxin binder contains natural hydrated sodium calcium aluminium silicates

^f^A combination of oxytetracyclin and neomycin at the concentration of 100 ppm (w/w)

^g^The diets were formulated using FeedLIVE International software (Thailand)

**Table 2:** Composition and nutrient content of finisher diets

Ingredients	Dietary treatment^a^							
	Negative control	Positive control	T1	T2	T3	T4	T5	T6
Corn	54.70	54.70	54.89	54.89	54.80	54.69	54.80	54.69
Soybean	29.10	29.10	27.04	27.04	29.30	29.31	29.30	29.31
Wheat pollard	5.36	5.35	5.90	5.90	3.41	3.16	3.41	3.16
CPO	3.460	3.460	3.400	3.400	3.74	3.815	3.74	3.815
Fish meal (55 %)	3.600	3.600	5.040	5.040	3.870	3.945	3.870	3.945
L-Lysine	0.25	0.25	0.25	0.25	0.25	0.25	0.25	0.25
DL-Methionine	0.20	0.20	0.20	0.20	0.20	0.20	0.20	0.20
Monodicalcium phosphate21	1.40	1.40	1.35	1.35	1.40	1.40	1.40	1.40
Calcium carbonate	1.30	1.30	1.00	1.00	1.30	1.30	1.30	1.30
Choline chloride	0.06	0.06	0.06	0.06	0.06	0.06	0.06	0.06
Salt	0.25	0.25	0.25	0.25	0.25	0.25	0.25	0.25
Mineral premix^b^	0.10	0.10	0.10	0.10	0.10	0.10	0.10	0.10
Vitamin premix^c^	0.06	0.06	0.06	0.06	0.06	0.06	0.06	0.06
Antioxidant^d^	0.01	0.01	0.01	0.01	0.01	0.01	0.01	0.01
Toxin binder^e^	0.15	0.15	0.15	0.15	0.15	0.15	0.15	0.15
Antibiotic^f^		0.01						
postbiotic RI11			0.30		0.30	0.30		
postbiotic RG14				0.30			0.30	0.30
Inulin					0.80	1.00	0.80	1.00
Calculated nutrient content (g/kg)^g^								
Crude protein	199.1	199.1	199.1	199.1	199.0	199.1	199.0	199.1
Metabolizable energy (MJ/Kg)	13.00	13.00	13.00	13.00	13.00	13.00	13.00	13.00
Calcium	10.3	10.3	10.0	10.0	10.5	10.5	10.5	10.5
Total phosphorus	8.1	8.1	8.2	8.2	8.0	8.0	8.0	8.0
Avail. P for poultry	4.7	4.7	4.9	4.9	4.7	4.7	4.7	4.7

^a^Negative control: (basal diet), Positive control: (basal diet + neomycin and oxytetracycline), T1: (0.3 % RI11), T2: (0.3 % RG14), T3: (0.3 % RI11 + 0.8 % Inulin), T4: (0.3 % RI11 + 1.0 % Inulin), T5: (0.3 % RG14 + 0.8 % Inulin), T6: (0.3 % RG14+ 1.0 % Inulin)

^b^Mineral mix contains Fe 100 mg, Mn 110 mg, Cu 20 mg, Zn 100 mg, I 2 mg, Se 0.2 mg, Co 0.6 mg

^c^Vitamin premix contains retinol 2 mg, cholicalciferol 0.03 mg, α-tocopherol 0.02 mg, menadione 1.33 mg, cobalamin 0.03 mg, thiamine 0.83 mg, riboflavin 2 mg, folic acid 0.33 mg, biotin 0.03 mg, panthothenic acid 3.75 mg, niacin 23.3 mg, pyridoxine 1.33 mg

^d^Antioxidant contains butylated hydroxyanisole (BHA)

^e^Toxin binder contains natural hydrated sodium calcium aluminium silicates

^f^A combination of oxytetracyclin and neomycin at the concentration of 100 ppm (w/w)

^g^The diets were formulated using FeedLIVE International software (Thailand)

### Timing of sample collection

On weekly basis, BW and feed intake (FI) were recorded and WG and FCR were calculated. For sampling, 12 birds per treatment group were slaughtered at day 42. FCR was calculated as follow: FCR = total feed consumed by birds/total weight gain.

### Faecal LAB, ENT count and pH determination

The method of Foo et al. [29] was used to determine the faecal LAB and population of ENT. Faecal samples were kept at room temperature for 1 h once the 10-fold dilution (w/v) was done in sterile peptone water. Furthermore, 10-fold serial dilutions (v/v) were done after the 1 h soacking time. MRS-agar (Lactobacillus-Agar MRS) (Merck, KgaA, Darmstadt) was used to perform the enumerations of LAB. Incubation of plates was performed in anaerobic jars for 48 h at 30 °C. The incubation of ENT was performed aerobically for 24 h at 37 °C after spreading and counting them on EMB-Agar (Eosin-methyleneblue Lactose Sucrose Agar, Merck, KgaA, and Darmstadt). The base 10 logarithm of colony-forming unit (CFU) (logCFU) per g was applied to express the number of CFU. The whole samples were in triplicates. Almost 9 ml of deionized distilled water was used to homogenise about 1 g of the sample in a universal tube. Mettler-Toledo pH meter with a glass electrode (Mettler-Toledo LTD, England) was used to measure the pH. The meter was calibrated prior to measuring the pH of the samples by using buffer solutions (Merck, KgaA, Dramstadt) at pH 4 and 7.

### Histomorphology

Specimens were taken from three different parts of jejunum, duodenum, and ileum. These specimens were taken from the following locations;

(i) the middle part of the duodenal loop,
(ii) midway between the end point of duodenal loop and Meckel’s diverticulum (jejunum), and
(iii) midway between the Meckel’s diverticulum and the ileo-caecal junction (ileum), and fixed in 10 % neutral buffered formalin.

A tissue processing machine (Leica, Japan) was used to excise, dehydrate these specimens. Then they were embedded in paraffin wax. Each sample was cut into sections (4 mm). These sections were stained with haematoxylin and eosin, fixed on slides, and then mounted and examined under the light microscope. The tip of the villus to the villus-crypt junction area was measured as the villus height. Furthermore, the crypt depth was defined as the depth of the invagination between two villi.

### Determination of VFA

The modified method of Thanh et al. [14] was applied to determine the VFA concentration in the faeces. From each sample, 1 g faeces (stored at −20 °C) were weighed. Then, 1 mL of 24 % metaphosphoric acid was added which was diluted in 1.5 M sulphuric acid (BDH Laboratories, Poole, UK). First, under room temperature, this mixture was stored overnight and then the mixture was centrifuged at 10 000 × g for 20 min at 4 °C. The supernatant was kept in a 1.5-ml screw-capped vial (Kimble Glass Inc., USA). For the GLC analysis, the internal standard, 20 mM 4-methyl-valeric acid (Sigma Chemical Co., St. Louis, MO, USA) was consistently added to the supernatant to make up 10 mM and the mixture was stored at −20 °C. The VFA was separated on a Quadrex 007 Series (Quadrex Corp., New Haven, CT 06525, USA). The bonded phase was a fused silica capillary column (15 m, 0.32 mm ID, 0.25 mm film thickness) with a 6890 N (Hewlett-Packard, Avondale, PA) equipped with a flame ionization detector. The carrier gas was purified liquid nitrogen with a flow rate of 60 mL/min. The temperature of the injector and detector was set at 230 °C. The column temperature was set at 200 °C. For identification of peaks, the commercial standards of 20 mM acetic, and 10 mM each of propionic, butyric, isobutyric, valeric, isovaleric and 4-methyl-valeric acids from Sigma were used as external standards.

### Total RNA isolation and reverse transcriptase polymerase chain reaction (RT-PCR) analysis of hepatic IGF1 and GHR

Lysis buffer (Qiagen, USA) received 30 mg (per sample) of frozen liver and it was properly homogenized. Following the manufacturer’s instructions, Qiagen one-step RT-PCR kit (Qiagen, USA) was used to perform the RT-PCR. Real- time RT qPCR analyses were done using QuantiTect Primer Assay (200) IGF1 (QT00621334), GHR (QT00601321) and B-actin (QT00600614). Thereafter, NanoDrop was used to evaluate the purity of RNA at 260/280 OD ratio and RNA integrity. Only highly purified samples (OD260/280 > 1.8) were chosen for further manipulation. The master mix preparation was also conducted following the manufacturer’s procedure. The total volume of the reaction was 25 μl for each gene of interest arranged as 2× QuantiFast SYBR Green RT-PCR Master Mix 12.5 μl, 1 μl of forward primer, 1 μl of the reverse primer, 0.25 μl QuantiFast RT Mix, 1 μl of RNA, and finally 9.25 μl of RNase-free water. The reaction was carried out in a Bio-Rad thermal cycler (MyCycler, Germany). The RT-PCR conditions were as follows: (1) reverse transcription, 30 min, 50 °C, (2) initial PCR activation step, 15 min, 95 °C, (3) 3-step cycling for 40 cycles, each cycle consisting of denaturation for 30 s at 94 °C followed by annealing for 30 s at 52–57 °C and extension for 1 min at 72 °C. The linearity of response was ensured and the saturation of the reaction was averted by optimizing the template concentration and the cycle number. For standardization of the expression data, the β-actin mRNA fragment was used as internal standard (housekeeping gene). The results were standardized to the levels obtained for the β-actin gene. It was done by taking the ratio of the value obtained for the gene of interest to that of β-actin and then relative to the control. 2^-ΔΔCt^ (ΔΔCt = ΔCt Test sample-ΔCt Calibrator sample) calculated the relative mRNA expression

### Statistical analysis

Data analysis was performed using the General Linear Model procedure of the Statistical Analysis System. Means were compared using the Duncan Multiple Range Test. The Bio-Rad CFX Manager 3.0 Software of the C1000 Touch thermal cycler-CFX96 Real time PCR (BIO-RAD, Foster city, California, USA) was used to calculate the relative gene expression of target genes in comparison to the β-actin reference gene.

## Results

### Growth performance

The growth performance of the birds fed diets containing different additives is presented in Table 3. Birds fed with T3, T4 and T6 had higher (*p* < 0.05) final BW and total WG than other treatments. The final BW and WG of birds fed the negative control diet, postive control diet, T1, T2 and T5 were similar (*p* > 0.05). There was no significant difference (*p* > 0.05) among the treatments for FI. Birds fed with T3 and T6 had lower (*p* < 0.05) FCR compared with birds fed the negative control diet. The FCR of birds fed T3, T4, T5, T6 and positive control were similar (*p* > 0.05). Similarly, the FCR of birds fed negative control, positive control, T1, T2, T4 and T5 diets did not differ.

**Table 3:** Growth performance at week 6 of treatments supplemented with different postbiotics and differen levels of inulin

Parameter	Dietary treatments^e^								SEM
	Negative control	Positive control	T1	T2	T3	T4	T5	T6	
FI (g)	4245.17	4153.71	4289.19	4298.08	4199.29	4279.61	4187.49	4267.91	25.03
BW (g)	2239.59^b^	2248.93^b^	2267.28^b^	2266.69^b^	2334.90^a^	2330.79^a^	2264.72^b^	2345.48^a^	6.51
WG (g)	2189.10^b^	2198.34^b^	2217.79^b^	2217.52^b^	2284.31^a^	2279.52^a^	2215.59^b^	2295.24^a^	6.50
FCR	1.94^a^	1.89^abc^	1.93^ab^	1.93^ab^	1.84^c^	1.88^abc^	1.89^abc^	1.86^bc^	0.01

^abc^means within a row for each parameter with different superscripts are significantly different (*p* < 0.05)

^e^negative control: basal diet, positive control: basal diet + neomycin and oxytetracycline, T1: (0.3 % RI11), T2: (0.3 % RG14), T3: (0.3 % RI11 + 0.8 % inulin), T4: (0.3 % RI11 + 1.0 % inulin), T5: (0.3 % RG14 + 0.8%inulin), T6: (0.3 % RG14 + 1.0%inulin)

### Faecal LAB, ENT and pH

**Fig. 1:**
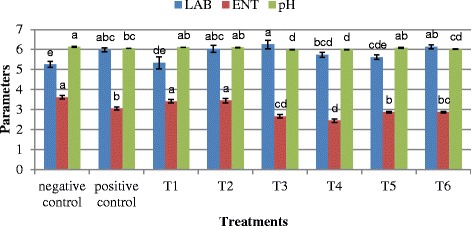
Microbiota counts (log CFU/g) and pH of broiler supplemented with different treatment diets at week 6. Treatments: Negative control: basal diet, Positive control: basal diet + neomycin and oxytetracycline, T1: (0.3 % RI11), T2: (0.3 % RG14), T3: (0.3 % RI11 + 0.8 % inulin), T4: (0.3 % RI11 + 1.0 % inulin), T5: (0.3 % RG14 + 0.8 % inulin), T6: (0.3 % RG14 + 1.0 % inulin). Bars with no common letter differ significantly (*p* < 0.05)

The faecal LAB, ENT and pH of birds fed various treatment groups are shown in Fig. 1. Significantly, the faecal pH for T3 were lower (*p* < 0.05) than the negative and positive controls. Dietary treatments affected (*p* < 0.05) LAB and ENT counts. Postbiotic and inulin increased (*p* < 0.05) faecal LAB and decreased ENT count when compared to negative control.

### Histomorphology

The villus height and crypt depth of the duodenum, ileum and jejunum of birds fed different dietary treatments are shown in Table 4. Birds fed T3 and T6 had significantly higher (*p* < 0.05) villus height in the duodenum than the other treatments. Birds fed T2, T3, T4 and T6 had higher villus height in the ileum than the negative control. However, there were no significant differences (*p* > 0.05) for villus height in jejunum, and crypt depth in duodenum, jejunum and ileum among the treatments.

**Table 4:** Villus height and crypt depth in small intestine of treatments supplemented with different postbiotics and different levels of inulin

Parameter	Dietary treatments^e^								SEM
	Negative control	Positive control	T1	T2	T3	T4	T5	T6	
Villi height week 6, μm									
Duodenum	1304.88^b^	1304.75^b^	1306.80^b^	1308.55^b^	1419.68^a^	1311.18^b^	1315.80^b^	1395.38^a^	9.08
Jejunum	837.75	875.34	887.85	894.15	942.60	911.90	845.60	943.07	8.36
Ileum	540.90^c^	595.71^ab^	562.90^bc^	617.30^a^	634.08^a^	610.87^a^	597.25^ab^	622.18^a^	7.29
Crypt depth week 6, μm									
Duodenum	168.12	165.52	177.85	166.00	174.95	143.17	170.22	173.72	3.78
Jejunum	132.75	139.20	143.07	127.85	139.12	128.85	120.72	134.85	3.34
Ileum	101.07	106.15	105.50	102.12	107.42	105.75	95.15	109.45	2.67

^abcd^means within a row for each parameter with different superscripts are significantly different (*p* < 0.05)

^e^negative control: basal diet, positive control: basal diet + neomycin and oxytetracycline, T1: (0.3 % RI11), T2: (0.3 % RG14), T3: (0.3 % RI11 + 0.8 % inulin), T4: (0.3 % RI11 + 1.0 % inulin), T5: (0.3 % RG14 + 0.8%inulin), T6: (0.3 % RG14 + 1.0%inulin)

### Volatile fatty acid

The faecal VFA of birds fed different dietary treatments is presented in Table 5. The result shows that acetic acid is the major VFA found in the broiler faeces followed by butyric and propionic acid. Brioler chickens fed T6 had the highest concentration of acetic acid and total VFA which was significantly different (*p* < 0.05) from other treatment groups. Birds fed T3 had the highest (*p* < 0.05) propionic acid as compare to birds fed with other treatments. No significant difference (*p* > 0.05) was observed for butyric acid in all treatment groups.

**Table 5:** Faecal VFA in broiler chickens fed with postbiotics and different levels of inulin

Parameter (mM)	Dietary treatments^e^								SEM
	Negative control	Positive control	T1	T2	T3	T4	T5	T6	
Acetic	27.78^c^	29.78^c^	30.39^c^	34.70^bc^	40.22^b^	31.47^bc^	36.85^bc^	49.30^a^	1.57
Propionic	0.27^c^	0.66^bc^	0.89^ab^	0.71^bc^	1.30^a^	0.84^ab^	0.87^ab^	0.66^bc^	0.07
Butyric	0.21	0.22	0.39	0.38	0.40	0.28	0.43	0.31	0.02
Total	28.25^d^	30.65^cd^	31.67^cd^	35.45^bcd^	41.92^b^	32.59^cd^	38.15^bc^	50.27^a^	1.59

^abcd^means within a row for each parameter with different superscripts are significantly different (*p* < 0.05). ^e^negative control: basal diet, positive control: basal diet + neomycin and oxytetracycline, T1: (0.3 % RI11), T2: (0.3 % RG14), T3: (0.3 % RI11 + 0.8 % inulin), T4: (0.3 % RI11 + 1.0 % inulin), T5: (0.3 % RG14 + 0.8%inulin), T6: (0.3 % RG14 + 1.0%inulin)

### IGF1 and GHR mRNA expression

**Fig. 2:**
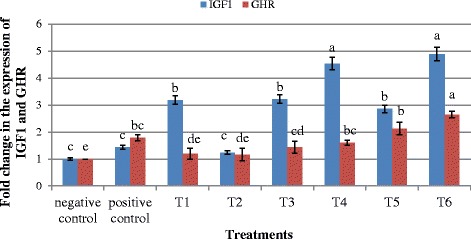
IGF1 and GHR mRNA expression in the liver of broiler chicken. Treatments: Negative control: basal diet, Positive control: basal diet + neomycin and oxytetracycline, T1: (0.3 % RI11), T2: (0.3 % RG14), T3: (0.3 % RI11 + 0.8 % inulin), T4: (0.3 % RI11 + 1.0 % inulin), T5: (0.3 % RG14 + 0.8 % inulin), T6: (0.3 % RG14 + 1.0 % inulin). Bars with no common letter differ significantly (*p* < 0.05)

Gene expression profile of liver fed different dietary treatments is shown in Fig. 2. Birds fed T6 had the highest (*p* < 0.05) IGF1 mRNA expression. The IGF1 expression in T1, T3, T4, T5 and T6 were significantly higher (*p* < 0.05) than the negative and positive control. The GHR mRNA expression in the liver of broilers fed with T6 was significantly higher (*p* < 0.05) than that of other treatment groups.

## Discussion

Postbiotics influenced the growth rate of broiler chickens. It has bacteriostatic and bactericidal ability which decreases the pathogenic bacterial load in gastrointestinal microbiota. Kareem et al. [19] reported that *L. plantarum* exhibited inhibitory effect against various pathogens. Chicory root powder which comprise 68 % inulin enhances food digestion and absorption through jejunum histomorphometry modification thereby improve growth performance in broiler chickens [30]. The current results are in agreement with those of Thanh et al. [14] who found that birds fed combinations of metabolites produced by *L. plantarum* had higher (*p* < 0.05) final BW and WG compared with those fed the negative control diet. In contrast, Rosyidah et al. [31] did not observe significant difference in BW and WG in broilers fed with metabolites and combination of metabolite and acidifier groups and those fed positive and negative control diets.

The similarity in the BW and WG of birds fed the negative control diet and postive control diet suggests that antibiotics are no longer effective as growth enhancer and may not be effective as antibacterial in the future. The current observation is in tandem with that of Aristides et al. [32] who observed that dietary supplementation of avilamycin did not affect WG in broiler chickens. Postbiotics added with inulin was even better than diet added with antibiotic in BW and WG.

The lower FCR in T3 and T6 birds compared with the negative control birds is consistent with the report of Liu et al. [33] who found that probiotic, prebiotic and synbiotic significantly improved feed efficiency as compare to the negative control diet. In contrast, Elrayeh and Yildiz [34] reported 0.7 % inulin had no effect on FCR of broiler chickens.

Different combinations of postbiotics and inulin decreased faecal pH and ENT count. This observation corroborates the report of earlier findings which showed that addition of prebiotics beneficially modified intestinal microbiota in animal models and human studies. The authors posited that addition of prebiotics enhanced the population of protective bacteria (i.e. *Lactobacilli* and *Bifidobacteria*) and hindered the attachment of pathogenic bacteria to the gut epithelium [35–37]. The current findings are in agreement with those of Loh et al. [10] who observed that dietary postbiotics increased the faecal LAB and reduced the faecal pH and faecal ENT in laying hens. Also, Rosyidah et al. [31] observed an increase in LAB count and a decrease in ENT count in the faeces of broilers fed with a metabolite produced by *L. plantarum*. Nabizadeh [38] found that dietary 1 % inulin reduced *E. coli* counts and pH in cecal contents in broiler chickens. Contrarily, Biggs et al. [39] found that corn-soybean meal diets containing 4 % inulin and some oligosaccharides had no effect on cecal *Lactobacillus, Bifidobacterium, Clostridium perfringens,* or *E. coli* populations in 21-d-old chicks. Abdel-Raheem et al. [40] also reported that prebiotic, probiotic and synbiotic had no effect on cecal *Lactobacilli* and *E. coli* counts in broilers chickens.

Villus height and crypt depth are reliable indicators of the gut function and health. According to Uni et al. [41] the health status of the gastrointestinal tract of an animal is a true reflection of intestinal morphology. Birds fed T3 and T6 had higher (*p* < 0.05) duodenal villus height than the control diets while birds fed T2, T3, T6 and T4 had higher (*p* < 0.05) ileal villus compared with the control diets. Our findings are consistent with the findings of earlier studies. The postbiotic and prebiotic altered the mucosal architecture in terms of longer villi and increased birds’ performance [14, 40, 42]. Furthermore, probiotics and synbiotics can enhance broiler performance by improving the intestinal morphology and microbial balance which are associated with suppressing intestinal pathogens such as *E. coli Campylobacter* and *Salmonela* and at the same time increase nutrient digestibility [43, 44]. In contrast, according to Nabizadeh [38], inulin did not affect the mophorlogy of duodenum, ileum and jejunum of broiler chickens.

The observed increase in the cocentration of acetic acid in T6, the higher propionic acid in T3 and increased in total VFA in both T3 and T6 compared with other treatments could be responsible for the increased lactic acid bacteria and decreased ENT counts and pH observed in the treatments. This finding is consistent with those of Loh et al. [10] and Thu et al. [16]. Postbiotics originating from *Lactobacillus* include valuable compounds such as organic acids and bacteriocin which enhance the growth of lactic acid bacteria [10]. Moreover, Van der Wielen et al. [45] reported that during growth of broiler chickens, VFA are responsible for the reduction in numbers of ENT in the ceca. In addition, the increased VFA could be due to the inulin as it contains polysaccharides and oligosaccharides. Gebbink et al. [46] demonstrated that fructooligosaccharide can be used to assist and maintain the healthy gastrointestinal tract environment by increasing the colonization of *Bifidobacteria* or reducting the *E. coli* in the intestinal system. Digestive enzymes do not hydrolyse the fructooligosaccharide in the small intestine of monogastrics and thus, it enters into the colon intact. Colonic microbiota metabolize it completly. Gases, lactate, and short cain fatty acid (i.e. acetate, propionate, and butyrate) are the output of carbohydrate fermentation [38]. The main fermentative chamber in broiler chicken is the caecum and this contain the largest number of bacteria compared with other gastrointestinal tract segment. Hence, the microbiota has high ability to ferment the carbohydrates [47].

Dietary postbiotic and inulin influenced the expression of mRNA IGF1 and mRNA GHR in the liver. The increase in IGF-I mRNA and GHR mRNA in birds fed T6 is a true reflection of the growth performance of the birds. It is improtant to note that the IGF1 level, feeding level, and growth rate are concurrent [22]. The dependence of nutritional and growth hormones on hepatic IGF1 production has been demonstrated [23, 25]. Moreover, Amongst the genes influencing growth, IGF1 has been demonstrated as an indicator of growth rate in chicken by several authors [48, 49]. The pituitary relseases the growth hormone which stimulates the hepatic production of IGF1 through the actions of GH activated GH receptors. However, the overall nutritional status of the animal modulated the ability of hepatic tissue to respond to GH [25]. The IGF1 level can be affected by factors and situations that affect primary processes and controll the IGF1 production. These results might provide bases for the development of IGF1 as a growth index. Results from this study corroborate the findings of Beckman et al. [50] who found significant and positive correlation between mean growth rates of juvenile Chinook salmon and mean plasma IGF1 levels. However, other studies have not demonstrated any relationship between IGF1 and growth [51, 52] which can lead to uncertainties about the consistency of IGF1 growth relationships.

## Conclusion

The study demonstrated that addition of postbiotics and inulin had beneficial effects on total BW, feed efficiency, mucosa architecture and expression of IGF1 and GHR mRNA in the liver of broiler chickens. However, birds fed T4: (0.3 % RI11 + 1.0 % inulin) and T6: (0.3 % RG14 + 1.0 % inulin) had higher total BW and feed efficiency than the other treatments. The faecal pH and ENT was reduced while VFA and LAB was increased in birds fed T4 and T6 as compared to the other treatments. Thus, both T4 and T6 could be used as a substitute for antibiotics in broiler diet to improve the growth and gut health in broiler chickens.

## Abbreviations

BW, body weight; CFU, colony-forming unit; ENT, *Enterobacteriaceae*; FCR, feed conversion ratio; FI, feed intake; GHR, growth hormone receptor; IGF1, insulin like grouth factor 1; *L. plantarum*, *Lactobacillus plantarum*; LAB, lactic acid bacteria; MRS, de-Mann Rogosa Sharpe; RT-PCR, reverse transcriptase polymerase chain reaction.; VFA, volatile fatty acid; WG, weight gain.
